# Characterization of *Planktochlorella nurekis* Extracts and Virucidal Activity against a Coronavirus Model, the Murine Coronavirus 3

**DOI:** 10.3390/ijerph192315823

**Published:** 2022-11-28

**Authors:** Jacqueline Graff Reis, Isabella Dai Prá, William Michelon, Aline Viancelli, David Guillermo Piedrahita Marquez, Caroline Schmitz, Marcelo Maraschin, Sidnei Moura, Izabella Thaís Silva, Geovanna de Oliveira Costa, Tiago Tizziani, Louis P. Sandjo, David Rodríguez-Lázaro, Gislaine Fongaro

**Affiliations:** 1 Laboratory of Applied Virology, Department of Microbiology, Immunology, and Parasitology, Federal University of Santa Catarina, Florianópolis 88040-900, SC, Brazil; 2 Department of Pharmaceutical Sciences, Federal University Santa Catarina, Florianópolis 88040-900, SC, Brazil; 3 Mestrado Profissional em Engenharia Civil, Sanitária e Ambiental, Universidade do Contestado Concórdia, Concórdia 89520-000, SC, Brazil; 4 Plant Morphogenesis and Biochemistry Laboratory, Federal University of Santa Catarina, Florianópolis 88034-000, SC, Brazil; 5 LBIOP—Laboratory of Biotechnology of Natural and Synthetics Products, Technology Department, Biotechnology Institute, University of Caxias do Sul, Caxias do Sul 95070-560, RS, Brazil; 6 Programa de Pós-Graduação em Química, Department of Chemistry, CFM, Federal University of Santa Catarina, Florianópolis 88040-900, SC, Brazil; 7 Microbiology Section, Faculty of Sciences, University of Burgos, 09001 Burgos, Spain; 8 Centre for Emerging pathogens and Global Health, University of Burgos, 09001 Burgos, Spain

**Keywords:** coronavirus, SARS-CoV-2, murine coronavirus, metabolomic approach, NMR biomarkers, UPLC-MS, viricidal activity, antiviral activity

## Abstract

Certain members of the Coronaviridae family have emerged as zoonotic agents and have recently caused severe respiratory diseases in humans and animals, such as SARS, MERS, and, more recently, COVID-19. Antivirals (drugs and antiseptics) capable of controlling viruses at the site of infection are scarce. Microalgae from the Chlorellaceae family are sources of bioactive compounds with antioxidant, antiviral, and antitumor activity. In the present study, we aimed to evaluate various extracts from *Planktochlorella nurekis* in vitro against murine coronavirus-3 (MHV-3), which is an essential human coronavirus surrogate for laboratory assays. Methanol, hexane, and dichloromethane extracts of *P. nurekis* were tested in cells infected with MHV-3, and characterized by UV-vis spectrophotometry, nuclear magnetic resonance (NMR) spectroscopy, ultraperformance liquid chromatography-mass spectrometry (UPLC-MS), and the application of chemometrics through principal component analysis (PCA). All the extracts were highly efficient against MHV-3 (more than a 6 Log unit reduction), regardless of the solvent used or the concentration of the extract, but the dichloromethane extract was the most effective. Chemical characterization by spectrophotometry and NMR, with the aid of statistical analysis, showed that polyphenols, carbohydrates, and isoprene derivatives, such as terpenes and carotenoids have a more significant impact on the virucidal potential. Compounds identified by UPLC-MS were mainly lipids and only found in the dichloromethane extract. These results open new biotechnological possibilities to explore the biomass of *P. nurekis*; it is a natural extract and shows low cytotoxicity and an excellent antiviral effect, with low production costs, highlighting a promising potential for development and implementation of therapies against coronaviruses, such as SARS-CoV-2.

## 1. Introduction

Coronaviruses (CoVs, subfamily Coronavirinae, order Nidovirales) are enveloped and positive-strand RNA viruses. The CoV virion has a triple protein membrane, containing spike proteins on the surface, responsible for binding of the virus to its receptor and cell entry [1,2,3]. CoVs are human and animal pathogens that cause respiratory, hepatic, and neurological problems [4,5] and have been responsible for epidemics and pandemics, such as severe acute coronavirus respiratory syndrome (SARS-CoV) in 2003, Middle Eastern coronavirus respiratory syndrome (MERS-CoV) in 2012, and SARS-CoV-2 coronavirus disease (COVID-19) in 2019 [5,6].

The Murine coronavirus (mouse hepatitis virus, MHV) has been used as a safe model (it does not infect humans) for SARS-CoV and SARS-CoV-2 because of the structural and genomic similarities between them. MHV causes various types of infection in mice: respiratory, hepatic, enteric, and neurological. Thus, the murine coronavirus has recently become widely used as a surrogate model for SARS-CoV-2 in prevention strategies, vaccine development, and new treatment methodologies [7,8,9].

Algae have been used to develop new medicines and treat viruses for many years [10]. Certain species, such as the microalga *Spirulina platensis*, have demonstrated an antiviral effect against adenovirus type 40 non-enveloped viruses [11] and proven antiviral effects against adenovirus type 7, adenovirus type 40, astrovirus type 1, coxsackievirus B4, and the rotavirus Wa strain, suggesting a strong potential of algae extracts as antivirals [12]. Several compounds obtained from microalgae, such as sulphated polysaccharides against influenza A [13] and EMCV [14] and phycobiliprotein against enterovirus 71 [15], have already been reported. Other studies have shown algae compounds to have much potential in therapeutic and prophylactic approaches against the group of viruses to which MHV-3 and SARS-CoV belong [16,17]. The antiviral activity of macroalgae extracts against members of the Coronaviridae family has been already reported, showing the ability to inactivate the virus by binding to the SARS-CoV spike glycoprotein and thus inhibiting viral entry into the cell, with minimal toxicity [16]. In addition to macroalgae, studies with microalgae have also reported an antiviral effect against respiratory viruses, such as adenovirus type 40 [11], adenovirus type 7 and adenovirus type 40 [12], influenza A (IAV), an enveloped virus [13], and influenza A and B viruses, RSV-A, and RSV-B [18].

Chlorellaceae is a family of unicellular green freshwater or marine microalgae commonly studied for a wide range of components that have antioxidant, antibacterial, antiviral, and antitumor potential [19,20,21]. The compounds formed by the unique metabolic pathways of microalgae arise from the processes that allow them to adapt to an environment in which they are subjected to a broad spectrum of stresses, such as variations in temperature, humidity, salinity, pressure, the availability of oxygen, and UV. These biotic and abiotic factors lead to differentiation in the production of compounds, such as carotenoids, fatty acids, proteins, and polysaccharides, which may vary between microalgae species and may also vary within the same community during the year depending on seasonal variation [22]. *Planktochlorella nurekis* is a coccoid shaped planktonic uninuclear organism consisting of vegetative cells with pot-shaped chloroplasts that contain pyrenoids and a cell wall composed of two layers [23].

We aimed to characterize the chemical profile of the dichloromethane, methanol, and hexane extracts obtained from *P. nurekis* and evaluate their biological activity against murine coronavirus (MHV-3) infection in vitro.

## 2. Materials and Methods

### 2.1. Obtention of the Microalgae Biomass

The microalgae used as inoculum was obtained directly from a facultative open pond used as tertiary treatment process to remove nutrients from a previously digested swine wastewater effluent. *P. nurekis* obtained from swine wastewater was grown in 10 L photobioreactors exposed to light (99 μmol m^−2^ s^−1^) under mixotrophic conditions (12 h:12 h, light:dark) with continuous agitation using a mechanical recirculation pump 1200 L.h^−1^ (Sarlobetter) at room temperature (23 °C). The photobioreactors were operated in a fed-batch mode using effluents from an anaerobic treatment system, which was diluted by adding 1.0 L to 6.0 L of chlorine-free tap water. The photobioreactors were inoculated with 70 ± 0.6 mg DW microalgae L^−1^. After 11 days, the biomass reached a fresh weight of 0.4 ± 0.1 g L^−1^. This biomass was then harvested by centrifugation at 3000× *g*, frozen immediately (−20 °C), and then lyophilized for further analysis and assays. In total, 20 g of *P. nurekis* biomass was exhaustively extracted with hexane, dichloromethane, and methanol at a 1:5 ratio (*w/v*; g:mL). The extracts were dried using a rotary evaporator maintained under vacuum at 50 °C and then stored at −20 °C).

### 2.2. Chemical Characterization of Planktochlorella nurekis Extracts

#### 2.2.1. Ultraviolet-Visible Spectroscopy (UV-VIS) Profile

Spectrophotometric assays were carried out using a SpectraMax 190 Microplate Reader (96-well flat bottom cell culture plate—Kasvi^®^). The dichloromethane, methanol, and hexane extracts were eluted four times and the absorbance spectra of the samples was measured at a range of wavelengths from 200 to 750 nm. The UV-vis scanning spectra (n = 3) were pre-processed using baseline correction, scatter correction, and standardization.

#### 2.2.2. Nuclear Magnetic Resonance Spectroscopy (NMR)

The three extracts were individually dried in a rotary evaporator and the drying completed by lyophilization for 24 h in a Labconco Freezone 4.5 plus instrument. The methanol sample was then suspended in 500 µL methanol-d4 and the dichloromethane and hexane extracts directly in CDCl_3_. The NMR experiments were performed on a Fourier 300 Bruker^®^ 9.4 Tesla (300 MHz for hydrogen frequency and 75.48 MHz for carbon frequency) spectrometer equipped with a 5 mm internal diameter BBI probe, with reverse detection and field gradient coils in the coordinate. The chemical shifts were determined relative to TMS as the internal standard and are expressed as δ values (ppm), with coupling constants reported in Hz. The spectra were acquired for the CDCl_3_ solutions using 5 mm quartz tubes at 303 K. For all analyses, 64 scans were used. NMR data acquisition and processing were performed using Bruker TopSpin™ software.

#### 2.2.3. Total Phenolic Content

One hundred mg of the three extracts were suspended and homogenized in 400 µL ethanol and 100 μL transferred to test tubes containing 75 μL Folin–Ciocalteu reagent. Subsequently, 875 μL of a 2% sodium carbonate solution (*w*/*v*) was added. The suspensions were vortexed for 1 min and the samples incubated for 1 h at room temperature in the dark. Then, the mixtures were transferred to the wells of a microplate and the absorbance at 760 nm measured using a SpectraMax 190 Microplate Reader. The stock standard solution for the calibration curve was prepared by appropriate dilution of the compound with methanol to provide a concentration range of 100 to 2500 μg/mL for gallic acid (y = 0.0005x + 0.023, R^2^ = 0.958). Results are expressed as mg/g of gallic acid equivalents (GAEs) [24].

#### 2.2.4. β-Carotene Content

The extracts of *P. nurekis* were suspended in 400 µL dichloromethane, vortexed for 1 min, and quantified using a SpectraMax 190 Microplate Reader at 470 nm. The stock standard solution was prepared by dilution of β-carotene with dichloromethane to provide a concentration range of 100 to 2500 μg/mL (y = 0.008x + 0.570, R^2^ = 0.980). Results are expressed as micrograms per gram of fresh weight of the sample [25].

#### 2.2.5. Amino Acids Characterization

Ten grams of *P. nurekis* biomass was subjected to acid hydrolysis with 100 mL 3N HCl for 24 h. Subsequently, the extract was filtered, rinsed with water, and centrifuged at 3000 rpm for 10 min until the removal of excess HCl. The filtered samples were lyophilized and 1 mL of the acid hydrolysate and 3 mL of a 0.2% *w*/*v* ninhydrin solution in 200 nM sodium phosphate buffer at pH 7.0 were transferred into an assay tube and the samples heated in a water bath for 30 min at 100 °C. Finally, the absorbance of the samples was measured at 570 nm with a spectrophotometer SpectraMax 190 Microplate Reader. The amino-acid concentration was predicted using calibration curves prepared with standard amino-acid solutions; curves were made to determine the concentration of cysteine, phenylalanine, histidine, isoleucine, proline, serine, threonine, tryptophan, and valine [26].

#### 2.2.6. Qualitative Analysis of the Extracts by Ultraperformance Liquid Chromatography-Mass Spectrometry (UPLC-MS)

Acetonitrile (ACN), water (LCMS grade, Sigma-Aldrich, St. Louis, MI, USA), and 85% p.a. grade formic acid (Vetec Química Fina, Rio de Janeiro, Brazil) were used for the (UPLC-ESI-MS) analyses. All solutions prepared for the UPLC analyses were filtered through a 0.22-µm hydrophobic membrane made of cellulose. Chromatographic analyses were performed on an Acquity H-Class UPLC-PDA system (Waters Co., Milford, AS, USA). An Acquity UPLC BEH C_18_ (50 × 2.1 mm i.d., 1.7 µm) column (Waters Co., USA) was used for the analysis. The column was maintained at 40 °C during the analyses. MS data were obtained using a quadrupole orthogonal acceleration time-of-flight (QTOF) mass spectrometer, Xevo GS-2 QTof, with an electrospray ionization (ESI) source, operating in both positive and negative modes, with the mass range between 100 and 1200 Da and a scan time of 1 s. The mobile phase system consisted of a gradient of 0.1% aqueous formic acid (pH 3.0) (A) and ACN (B) at a flow rate of 0.3 mL/min. The gradient consisted of 0–2 min, 90% A/10% B; 2–10 min, 55% A/45% B; 10–15 min, 10% A/90% B; 15–20 min, 90% A/10% B. The injection volume was 2 μL. The instrument settings in positive mode were a capillary voltage of 3.0 kV, sampling cone voltage of 40 V, source offset voltage of 80 V, desolvation temperature of 200 °C, source temperature of 80 °C, cone gas flow of 50 L/h, and desolvation gas flow of 500 L/h. Nitrogen was used as the nebulizer gas and argon as the collision gas. MS and MSE data (in two scan functions) were acquired in the centroid mode and monitored with a scan time of 1 s. The collision energy was 6 eV in function 1 and ramped from 25 to 35 eV in function 2. To assure accurate mass values, data were corrected during acquisition by an external reference (LockSprayTM) named leucine-enkephalin solution (1 ng/mL) at a flow rate of 20 μL/min. System control and data processing were performed using MassLynx 4.1 software (Waters Co., USA). All samples were prepared by dissolution of each extract in water: acetonitrile (9:1, *v*/*v*) to reach a concentration of 800 μg/mL.

### 2.3. Cytotoxicity Assay

Mouse fibroblast cells (L929, ATCC^®^ CCL-1) were maintained in Minimum Essential Medium (MEM; Thermo Fisher Scientific, Warsaw, Poland) supplemented with 10% heat-inactivated fetal bovine serum (Thermo Fisher Scientific, Poland), seeded into plates (96-well plate format, 2.5 × 104 cells/well), and maintained for 24 h at 37 °C in an atmosphere containing 5% CO_2_. Cells were exposed to various concentrations of the samples (48 µg/mL to 500 µg/mL of *P. nurekis* hexane, dichloromethane, and methane extracts) for 48 h under the same conditions of cell culture. Cell viability was assessed by the sulforhodamine B assay, which measures total protein mass [27]. The percentage of viable cells was plotted against each sample concentration. The CC_50_ values (the concentration of each extract that reduced cell viability by 50%) were calculated based on concentration-response curves using GraphPad Prism 8.0 (Graph Pad Software 8.0.0 version, La Jolla, CA, USA).

### 2.4. Virucidal Assay

This assay followed EN 14476:2013+A2:2019 for the evaluation of virucidal activity. Infectious virus titres were measured in plaque-forming units (PFU) as described by EN 14476:2013+A2:2019. Various amounts of virus (10^6^, 10^5^, 10^4^, 10^3^, 10^2^ and 10^1^ PFU) were incubated with various dilutions of extracts of *P. nurekis* ranging from 0 to 50 μg/mL (1:2 serial dilutions) for 15 min at 37 °C or 24 °C. Then, the reaction was blocked with 10% fetal bovine serum, and 400 µL of each test dilution added to L929 cells previously prepared in 24-well plates. After 1 h of contact with the cells, the inoculum was removed and the cell monolayer washed with phosphate-buffered saline. The cell cultures were then covered with MEM containing 1.5% carboxymethylcellulose (CMC; Sigma Chemical Co., St Louis, MO, USA) and incubated for 72 h at 37 °C in an atmosphere containing 5% CO_2_. The reduction of the virus was determined by the number of PFU relative to that of the untreated viral controls.

### 2.5. Statistical Analysis

The average and standard deviation were determined for each variable. The metabolite dataset and virus reduction were analyzed by analysis of variance (ANOVA) and the post hoc Tukey test. Principal component analysis (PCA) was also performed to investigate sample grouping and similarities and to identify the variables that most strongly influenced the classification of the *P. nurekis* extracts. Algorithms for the statistical analysis were developed using the MATLAB program (version 7.12.0.635) [28].

## 3. Results and Discussion

### 3.1. Chemical Characterization of Planktochlorella nurekis Extracts

#### 3.1.1. UV-VIS Profile

**Figure 1:**
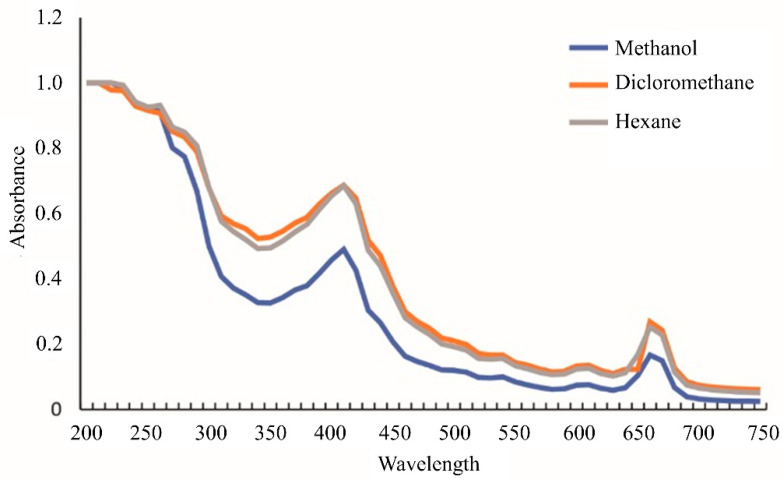
Spectral profiles of UV-Vis (λ = 200–750 nm) absorbances of methanol, dichloromethane, and hexane extracts (n = 3).

The extract of *P. nurekis* showed UV-VIS absorption profiles similar to those of other members of the taxonomic group following the same typical green algae extraction procedures [29]. The absorbance profiles showed different peak intensities for the three organic solvent extracts (Figure 1). The extracts were analyzed by UV-VIS spectroscopy over a range of 200 to 750 nm. No peaks were identified in the UV region, but rather a plateau between 240–260 nm, with a lower intensity peak at 280 nm. Maximum absorbance in the PAR region was detected at wavelengths of 410 nm and 660 nm.

The UV-VIS profile was used to compare the solvents of different polarity to identify possible differences in the spectral pattern. Despite the dichloromethane spectrum showing the highest intensities, it was impossible to identify distinct peaks between the extracts. In the UV spectral range, there was a band, with the absorbance peaks forming a plateau. In the PAR region, the extracts exhibited two significant absorption peaks at 460 nm and 660 nm, indicating the presence of carotenoids and chlorophyll compounds [29,30,31].

#### 3.1.2. β-Carotene, Phenols, and Amino Acids Content

The results of the tests for β-carotene, phenols, and amino acids of the *P. nurekis* extracts are presented in Table 1. The dichloromethane extracts showed a higher concentration of carotenoids (16.6 µg/g) than the hexane (10.1 µg/g) and methanol (7.14 µg/g) extracts. The polarity of the solvent had a positive effect on the concentration of total polyphenols. The most polar solvent, methanol, extracted more than twice the amount than the most nonpolar solvent, hexane. The concentration of polyphenols in the methanol, dichloromethane, and hexane extracts was 84 mg/g, 40 mg/g, and 29 mg/g, respectively. The amino acid that showed the greatest difference between the solvents was valine, with a concentration of 119 mg/g in the hexane extracts, which dropped to 54 mg/g in methanol. The other quantified amino acids did not show significant differences between the solvents. In a previous study using *P. nurekis*, the most abundant amino acids found were glutamic acid (56 mg/g), aspartic acid (50 mg/g), alanine (45 mg/g), leucine (45 mg/g), valine (32 mg/g), and arginine (30 mg/g) [32]. However, quantified amino acids were not an appropriate biochemical marker in this study because our data were very different from the pattern found in the literature, a factor that may have resulted from the culturing of this microalgae in swine wastewater, as it contains a high concentration of essential components that promote algal growth, such as carbon, nitrogen, and phosphorus [33].

Quantitative data on carotenoids showed a tendency towards greater extraction of these pigments by dichloromethane. This effect is influenced by several variables, such as the solubility of the molecules in the extractor solvent, the polar character of the extracted molecules and the permeability of the cell membrane, and the transport of this analyte in the cytoplasm [34]. The prominent carotenoids are lutein (polar) and β-carotene (relatively nonpolar) [35]. The solubility of these two carotenoids is higher in dichloromethane, which explains the tendency towards greater extraction of these analytes by this solvent [36]. Lutein showed low solubility in hexane (approximately 20 mg/L) and β-carotene low solubility in methanol (approximately 10 mg/g). Thus, a solvent of intermediate polarity that solubilizes the molecules is best suited for this purpose.

The phenolic compounds showed a close relationship with the polarity of the solvent, with the highest concentration being seen in methanol. The low concentration of phenolic compounds in nonpolar solvents was already expected, as polar substances need polar solvents to be eluted [37,38].

#### 3.1.3. NMR Profile

Results of the NMR analysis are presented in Supplementary Materials Figure S1 and the identified metabolites are listed in Table 2. The NMR spectra shown here are the first such results for the microalgae *P. nurekis* to be reported in the literature. The data were compared with previous reports in the literature and online databases, such as the Human Metabolome Database (HMDB), the Biological Magnetic Resonance (BMR) database, and PubChem.

The NMR profile showed the dichloromethane extract to have the highest metabolite concentration, followed by the hexane extract; the number of peaks in methanol was lower than for other solvents and were also less intense. The largest peaks in the methanol extract were located at 1.23 ppm and 3.16 ppm, which have been linked to fatty acids, such as linolenic acid. The other distinctive peaks in the sample, such as those located between 0.83 ppm and 0.95 ppm, indicate the presence of palmitic acid and isoleucine. Other peaks found at 1.90 ppm and 5.32 ppm confirm that the *P. nurekis* methanol extracts contained a significant amount of canthaxanthin and linoleic acid. However, these peaks were small relative to the those of the other main metabolites in the sample, confirming that the samples contained only low concentrations of these compounds [39]. The metabolites of the extract of *P. nurekis* in methanol were not quantified because they were below the limit of detection (LOD).

Both the dichloromethane and hexane extracts shared peaks at several frequencies, such as those found at 0.98 ppm, which signals the presence of vitamin B5, 1.26 ppm, which belongs to zeaxanthin, 3.50 ppm, which is associated with valine and violaxanthin, and 5.37 ppm, indicating common metabolite or bioactive groups found in the two extracts. The hexane sample had a distinctive peak at 2.32 ppm, which signals the presence of vitamin B2; whereas the dichloromethane extract had distinct peaks between 1.64 ppm, indicating the presence of lutein, 2.05 ppm, associated with the allylic protons on unsaturated fatty acids, 2.81 ppm, belonging to oleic acid, and 2.83 ppm, which indicates the presence of asparagine [40,41].

One of the main differences found between the dichloromethane and hexane extracts concerned the organic acids present in the two samples. First, the dichloromethane extract had a higher concentration and higher amounts of the already mentioned metabolites; only EDTA and isobutyric acid were present in higher concentrations in the hexane extract than in the dichloromethane extract. Isobutyric acid was the predominant acid in the hexane extract and the only secondary metabolite present at a higher concentration than in the dichloromethane extract. The hexane extract also contained organic substances, such as citric, acetic, formic, L-lactic, and 3-hydroxybutiric acid, but the dichloromethane extract contained a much higher concentration of these compounds. In addition, the bioactive compounds pyruvic, pyroglutamic, and succinic acid were found in the dichloromethane extract but not in the hexane extract.

On the other hand, both extracts contained alcohols, such as ethanol, glycerol, and 1,5-anhydrosorbitol. The hexane extract contained a higher concentration of these low molecular weight metabolites. Alcohols have a highly negative effect on metabolism, especially glycerol in microalgae. It has been reported that this compound inhibits the production of three metabolites, such as glucose-6-phosphate, fructose-6-phosphate, and glyceric acid-3-phosphate, key intermediates in carbon metabolism [42]. In addition to alcohols, the hexane extract contained higher concentrations of dimethyl sulfone and creatinine than the other extracts. These metabolites are less polar than the carboxylic compounds, which were predominant in the dichloromethane extract, and nonpolar solvents have a higher affinity for both metabolites. Nonpolar solvents, such as dichloromethane, elute nonpolar substances in a more significant proportion but elute medium polarity substances in smaller proportions. The virucidal efficiency of extracts with a more polar character is associated with the action of polar substances that bind to viral capsids, blocking the binding to proteins of the host cells [43]. Similar results were observed in a previous study in which dichloromethane extracts from green algae showed excellent antiviral activity against equine rhinopneumonitis, highlighting the potential of dichloromethane extracts as a source of molecules from green algae with virucidal and antiviral activity [44].

The signals for the amino acids were seen in the NMR spectra and helped us to reinforce the data obtained by amino-acid characterization. The peaks at δ = 0.99–1.04 ppm are related to valine and the multiplet between δ = 1.24 and 1.40 ppm indicates the presence of proline, isoleucine, and leucine protons. Except for alanine, which was not found in the dichloromethane extract, all the amino acids identified in NMR analysis were found in higher concentrations in dichloromethane extract. For example, amino acids such as L-threonine, L-tyrosine, L-leucine and L-methionine were present in a much higher proportion in the dichloromethane extract than the hexane extract, whereas L-glutamic acid was found at a similar concentration in both nonpolar solvents. These results indicate that *P. nurekis* is rich in amino acids, as already reported for other *Chlorella* sp., such as *Chlorella vulgaris*, for which leucine, phenylalanine, and tryptophan have been found [39]. Other amino acids, such as L-glutamic acid and alanine, have been found in *Chlorella* sp. [45]. One amino acid that has not yet been reported as a biomarker in microalgae is L-glutamine. Due to its high polarity, it was found at a higher concentration in dichloromethane and at a higher value than glutamate, indicating low N-stress, which has an impact on the concentration of carotenoids and isoprene derivates. A higher proportion of glutamate indicates higher nitrogen stress, resulting in a higher concentration of fatty acids, terpenic compounds, and carotenoids [46]. According to the literature, glutamine is used by microalgae to produce nucleosides and deoxy/ribonucleotides by the pyrimidine metabolism pathway [47]. Nonetheless, it will be necessary to analyze more compounds to draw a more solid conclusion.

Another indicator of the pyrimidine metabolism pathway found in the NMR spectra aside from glutamine was the presence of hypoxanthine. This metabolite is important due to its role in microalgae metabolism as a precursor of xanthine, which can be used to synthesize bioactive substances, such as astaxanthin [48], theobromine, and other methylxanthines, which have been reported to be antiviral agents against numerous pathogens, including SARS-CoV-2 [49].

In vitro studies on the activity of siphonaxanthin against SARS-CoV-2 in HEK293 cells overexpressing angiotensin-converting enzyme 2 (ACE2) showed the carotenoid to be the main bioactive compound with an antiviral action [50]. The authors suggested that the compound interferes with the interaction between the virus and the cells by binding to the S-glycoprotein [50]. Crocin has been shown to induce the downexpression of ACE2 expression and can reduce virally induced oxidative stress [51]. Finally, there is little information related to hypoxanthine and its activity against SARS-CoV-2, but it has been documented that hypoxanthine is a counterpart of the antiviral Favipavir, which inhibits the viral RNA polymerase and is effective against several strains of influenza viruses. Furthermore, it has recently been tested with other drugs, such as tocilizumab and oseltamivir, for the treatment of COVID-19 [52].

Butanol derivatives, such as 3-hydroxybutyrate, and isobutyric acid, were present in both extracts. These compounds can be produced by various microalgae from glucose and xylose. The polyhydroxyalkanoates (PHAs) in microalgae are obtained by the conversion of carbohydrate to 3-phosphoglycerate (PGA), followed by three possible pathways: the Entner–Doudoroff (ED) pathway, glycolysis, or the pentose phosphate pathway. These three pathways lead to the formation of pyruvate, which is transformed into acetyl-CoA, leading to the synthesis of butyrates by the PHA synthetic pathway [53].

Our samples were rich in fatty acids, such as oleic acid, linoleic acid, and alpha-linolenic acid. Those three lipids have also been found in microorganisms such as *Chaetoceros calcitrans* [54]. The peak at 2.05 ppm that appeared in the nonpolar extracts has also been found in the extracts of microalgae such as *Thalassiosira weissflogii*, *Cyclotella cryptica,* and *Nannochloropsis salina*. This peak is associated with palmitic acid and n-3 omega polyunsaturated fatty acids (PUFAs), such as alpha-linolenic acid. The presence of these metabolites will need to be confirmed by further tests [55]. Our results indicate a relationship between the amount of lipidic compounds extracted and the solvent used. The solvent with intermediate polarity allowed the best isolation of lipids due to the existence of an aliphatic and oxygenated region in the studied metabolite.

The peaks at δ = 0.50–2 ppm that were useful in identifying aliphatic compounds have also been observed for microorganisms such as *Chlorella* MAT-2008 [56] and *Chlorella* sp. [45]. Thus, the protonic NMR technique allows the identification of various metabolites and provides an initial approach for the quantification of specific bioactive lipids, such as fatty acids and polar lipids.

The NMR data showed the *P. nurekis* extracts to be rich in polar lipids, such as acylglycerols, xanthophylls, glycolipids, and phospholipids. In addition, there was a significant presence of carbohydrates and polyphenols. The signals for cholesterol are seen at δ = 0.83 ppm −0.95 ppm and 1.90 ppm, suggesting the presence of compounds such as zeaxanthin, lutein, and violaxanthin, which are commonly found in green plants and green microalgae [54].

Peaks between 3.16 and 5.32 ppm suggest the presence of carbohydrates, but more data are needed for confirmation. It will be necessary to carry out FT-IR and, in particular, MS analysis in which the main objective is the targeted study of the profile of polyphenols, carbohydrates, and lipids, focusing on the presumed metabolites that have been reported in the literature to have a virucidal effect. In the literature, both metabolites show similar peaks and despite the high concentration of phenolic derivatives, there appears to be a significant quantity of bioactive saccharides between 2.0 ppm and 4.0 ppm [40].

### 3.2. Analysis of the Extracts by UPLC-MS

The fractions from the hexane and dichloromethane extractions displayed almost the same UPLC-ESIMS profile (Supplementary Materials—Figures S2 and S3). Hexane showed the presence of 11 secondary metabolites and dichloromethane 18 in their chromatogram spectra (Supplementary Materials—Table S1).

The base peak ion (BPI) chromatogram of both extracts showed a peak at tR 4.53 min, with mass values of *m*/*z* 531.2817 [C_25_H_42_O_9_ + HCO_2_]^−^ and *m*/*z* 509.2745 [C_25_H_42_O_9_ + Na]^+^ in the negative and positive ionization modes, respectively. No fragmentation was observed in the negative ionization mode, whereas fragment ions of *m*/*z* 469.2790, *m*/*z* 325.2374, and *m*/*z* 233.1918 were obtained in the positive mode analysis. The precursor *m*/*z* 509.2745 produced *m*/*z* 469.2790 after losing NaOH (40 Da). The ion *m*/*z* 325.2374 appeared to be an in-source fragment formed by the of loss of a sugar moiety (162 Da) from [M+H]^+^. Cleavage of the ester function provided the fragment ion *m*/*z* 233.1918, corresponding to the fatty acyl cation. Based on these data, the structure of this metabolite was assigned to be (7Z,10Z,13Z)-2-hydroxy-3-(β-D-galactopyranosyl)oxy)propyl hexadeca-7,10,13-trienoate. The fragmentation pattern of compound 1 was consistent with that reported by Guella et al. [57].

Compounds 2 and 3 were identified as 13-hydroxyoctadecatrienoic acid and vernolic acid, respectively, with a *m*/*z* 293.2130 [C_18_H_30_O_3_-H]^−^ and 295.2289 [C_18_H_32_O_3_-H]^−^. Compound 2 represents a group of metabolites called oxylipins, which are widespread in algae [58].

Compounds 4 to 8 detected at tR 6.29, 7.39, 8.42, 9.59, and 9.89 min, respectively, yielded no fragments. However, their mass values of *m*/*z* 249.1852 [C_16_H_26_O_2_-H]^−^, 277.2180 [C_18_H_30_O_2_-H]^−^, 279.2330 [C_18_H_32_O_2_-H]^−^, 255.2332 [C_16_H_32_O_2_-H]^−^, and 281.2485 [C_18_H_34_O_2_-H]^−^ correspond to the structures of hexadecatrienoic acid, linolenic acid, linoleic acid, palmitic acid, and oleic acid, respectively. These fatty acid derivatives have been previously reported to be present in microalgae species from the genera *Aphanizomenon, Botryococcus, Chaetoceros, Chlorella, Cylindrotheca, Crypthecodinium, Isochrysis, Haematococcus, Dunaliella, Neochloris, Nostoc, Nannochloropsis, Pavlova, Phaeodactylum, Porphyridium, Arthrospira, Schizochytrium,* and *Thalassiosira* [59]. The dichloromethane extract contained all these fatty acids, whereas hexadecatrienoic acid and palmitic acid were not found in the hexane extracts. A study that used a mixture of vegetable oils composed mostly of fatty acids demonstrated the effectiveness of these compounds against SARS-CoV-2 and influenza viruses in an in vitro study [60]. The authors suggested that the antiviral action was related to the fatty-acid molecules, hypothesizing that the compounds penetrate and break down the lipid coat that covers enveloped viruses, thus deactivating the viruses at their point of entry.

Compound 8 was found at tR 3.72 min in the positive ionization mode and present only in the dichloromethane extract. Its mass value of *m*/*z* 181.1233 corresponds to the molecular formula [C_11_H_16_O_2_^+^H]^+^. This precursor was different from its fragment ion *m*/*z* 163.1130 by 18 Da, suggesting the loss of H_2_O. A literature search on this molecular formula led to a structure related to 4,4,7a-trimethyl-5,6,7,7a-tetrahydrobenzofuran-2(4H)-one, previously identified in seaweed [61].

The structure of a fatty acid found in the dichloromethane extract, namely stearidonic acid, was assigned to compound 10 detected at tR 4.97 min with a *m*/*z* 277.2166 [C_18_H_28_O_2_^+^H]^+^. Compound 11, detected at tR 5.48 min with a *m*/*z* 577.1348 [C_30_H_24_O_12_^+^H]^+^ produced a fragment ion of *m*/*z* 385.0938. A literature search showed compound 11 to be related to protocyanidin A1. The formation of the ion of *m*/*z* 385.0938 occurs after the cleavage of the CarO-Cacetal bond with hydrogen migration followed by RDA opening of ring C and the loss of ketene (42 Da). Thus far, only flavonoids and phenolic derivatives have been identified and reported in algae and microalgae [62]. Therefore, compound 11, which was found only in the hexane extract, is an unprecedented finding in microalgae metabolites. Compound 12 was detected in both the hexane and dichloromethane extracts at tR 6 min, with a mass value of *m*/*z* 301.1416, corresponding to [C_16_H_22_O_4_+Na]^+^. In its tandem mass spectrum, this metabolite showed two fragment ions of *m*/*z* 205.0866 and 149.0244 resulting from the elimination of sodium butanolate (96 Da) and sodium butanolate and but-1-ene (56 Da), respectively. Compound 13, found in dichloromethane at tR 8.35 min with a *m*/*z* 637.3063 [C_29_H_48_O_15_^+^H]^+^, produced fragment ions of *m*/*z* 581.2450, 525.1830, 495.26262, and 469.1125 in its tandem mass spectrum; these ions correspond to the sequential loss of four units of prop-1-en-1-one (56 Da). Based on these data, the structure of compound 13 was assigned to be related to O-tetrapropanoyloctanoate of sucrose.

Compound 14, observed at tR 8.35 min with a *m*/*z* 331.2852, produced two fragment ions in its tandem mass spectrum of *m*/*z* 313.2727 and 239.2368. The precursor is different from the first fragment by 18 Da, suggesting the loss of H_2_O, whereas the lighter fragment corresponds to the cation hexadecylidyneoxonium formed after glycerol (92 Da) elimination.

Compound 15, obtained at tR 9.01 min showed the precursor *m*/*z* 593.2747 [C_34_H_40_O_9_^+^H]^+^ and the fragment ion *m*/*z* 533.2573 in its mass spectra. Both ions differed by 60 Da, suggesting the loss of acetic acid. The structure was not identified because of the lack of diagnostic fragments.

Compound 16 was also identified as another monoglyceride, namely glyceryl stearate. It was detected at tR 10.47 min with a *m*/*z* 359.3179, corresponding to the molecular formula [C_21_H_42_O_4_^+^H]^+^. This precursor ion lost a molecule of H_2_O (18 Da) and glycerol (96 Da) to produce ions of *m*/*z* 341.3074 and 267.2713 (fatty acid moiety), respectively. Monoglycerides represent one of the valuable chemical constituents of microalgae used to produce bioenergy and their identification in this biomass has been well-documented [63]. The metabolite (17) at tR 11.13 min with a *m*/*z* 551.4241 [C_40_H_54_O+H]^+^ did not produce fragment ions in its MS/MS spectrum. However, a literature search using its molecular formula led to the structure of echinenone, a carotenoid previously identified in the microalgae *Scenedesmus obliquus* [64]. Compound 18 showed the same fragmentation pattern as compound 15 by losing only 60 Da, corresponding to acetic acid. Nevertheless, its structure could not be assigned because of low fragmentation. Compound 19 was identified as dioctyl phthalate. It was found at tR 11.94 min with a *m*/*z* 419.2663, corresponding to [C_24_H_38_O_4_+Na]^+^. This precursor gave the daughter ions *m*/*z* 301.1410 and 149.0244 in its MS/MS spectrum after losing oct-1-ene (112 Da) and sodium octanolate (272 Da). The methanol extract was more sensitive to the LC-MS analysis performed in the negative ionization mode (Supplementary Materials—Figure S4).

Compound 20 was detected at tR 7.98 min with a *m*/*z* 693.3342 [C_31_H_52_O_14_^+^HCO_2_]^−^ and gave the ion *m*/*z* 647.3315 [M-H]^−^ and the fragment *m*/*z* 249.1852 in its MS/MS spectrum, corresponding to hexadecatrienoic acid. This information led assignment of 3-hydroxy-2-[[(7Z,10Z,13Z)-1-oxo-7,10,13-hexadecatrienyl]oxy]propyl 6-O-β-D-galactopyranosyl-β-D-Galactopyranoside. Compound 21 was different from compound 20 by 164 Da, corresponding to the absence of a hexose (162 Da) and the presence of a double bond equivalent (2 Da). The tandem mass of compound 21, identified as 3-hydroxy-2-[[-1-oxo-4,7,10,13-hexadecatetraenyl]oxy]propyl β-D-Galactopyranoside, produced the ion *m*/*z* 247.1671, assigned as hexadecatetraenoic acid. This fragmentation pattern was consistent with those reported in the literature [57]. This ion was formed after the precursor lost the hexose and glycerol moieties. Two isomeric metabolites (22 and 23) with a structure related to 3-hydroxy-2-[[-1-oxo-7,10,13-hexadecatrienyl]oxy]propyl β-D-galactopyranoside were obtained at 8.49 and 8.71 min with the same mass value of *m*/*z* 531.2817 [C_25_H_42_O_9_+HCO_2_]^−^. These compounds differ from compound 20 by a hexose unit (162 Da). After losing the sugar and glycerol, an ion of *m*/*z* 249.1852, corresponding to the fatty acid chain, was formed. Compound 24, identified as 2-hydroxy-3-[[(9Z,12Z,15Z)-1-oxo-9,12,15-octadecatrien-1-yl]oxy]propyl6-O-β-D-galactopyranosyl-β-D-Galactopyranoside, at tR 8.93 with a mass value of *m*/*z* 721.3671 [C_33_H_56_O_14_+HCO_2_]^−^, produced a fragment ion of *m*/*z* 277.2180 after losing two hexoses and glycerol. Compound 25, identified as (2S)-2-hydroxy-3-[[(7Z,10Z)-1-oxo-7,10-hexadecadien-1-yl]oxy]propyl β-D-galactopyranoside, was heavier than compound 23 by 2 Da, suggesting the presence of less unsaturation. Their fragmentation pattern was identical, with the production of an ion corresponding to the fatty acid with a mass of *m*/*z* 251.2029. As found in the literature, such metabolites have been previously reported in the microalga *Chlorella sorokiniana* [65]. Another glycosylated monoglyceride was identified at tR 10.03 with a mass of *m*/*z* 699.3804 [C_31_H_58_O_14_+HCO_2_]^−^. A fragment ion was observed in its MS/MS spectrum of *m*/*z* 255.2332, corresponding to the ion palmitate. This information led to the assignment of 1-O-palmitoyl-3-O-[α-D-galactopyranosyl-(1→6)-β-D-galactopyranosyl]-sn-glycerol, previously reported in microalgae.

Compounds 27 and 28 gave ions of *m*/*z* 481.2598 [C_29_H_38_O_6_^-^H]^−^ and 483.2742 [C_29_H_40_O_6_^-^H]^−^, respectively. A literature search led to the structures of steroids related to poly-oxygenated ergosterol. Their structures could not be elucidated because of their low fragmentation. Compounds 29–32 were identified as derivatives of linolenic, linoleic, palmitic, and oleic acids.

Compound 33 with a tR at 14.73 min and *m*/*z* 953.5460 [C_49_H_80_O_15_^+^HCO_2_]^−^ was identified as disaccharide diglyceride. It produced two fragment ions in its tandem mass spectrum, assigned as the ions hexadecatrienoate (*m*/*z* 249.1852) and octadecatrienoate (*m*/*z* 277.2180). On the basis of this information, the structure of compound 33 was assigned as [[-1-oxo-9-hexadecatrien-1-yl]oxy]-3-[[-1-oxo-octadecatrien-1-yl]oxy]propyl 6-O-α-D-galactopyranosyl-β-D-galactopyranoside. Hexosyl mono- and diglycerides have been previously reported to be present in microalgae, supporting our finding [66].

The compounds identified in the UPLC-MS analysis were mostly associated with the lipid class, in which palmitic and stearidonic acid stood out as being present only in the dichloromethane extracts, which presented greater virucidal activity. Our results show that there is a relationship between the number of lipidic compounds extracted and the solvent used. The solvent with intermediate polarity was the one that allowed the best isolation of lipids, due to the existence of an aliphatic and oxygenated region in the metabolite.

### 3.3. Cytotoxicity of Planktochlorella nurekis

For the cytotoxic evaluation, all samples were tested on L929 cell line for 48 h and stained with SRB that binds stoichiometrically to cell proteins and then can be extrapolated to measure cell viability. The CC_50_ values were calculated for each sample and are summarized in Table 3. All the samples showed low cytotoxicity, and the dichloromethane extract presented an essential cytotoxic effect on L929 cells with a CC_50_ value of 53.19 µg/mL.

### 3.4. Virucidal Activity of Planktochlorella nurekis

The viricidal activity of the *P. nurekis* extracts is summarized in Table 4. Treatment with the microalgae methanol extract resulted in a reduction of coronavirus MHV-3 infection of 6 Log10 PFU and 8 Log10 PFU at 24 ± 2 °C and 35 ± 2 °C, respectively, regardless of the extract concentration (Table 4). Treatment with the hexane extract resulted in a reduction of 7 Log10 PFU (12.5 to 50 µg/mL) at 24 °C, but there was no reduction in coronavirus MHV-3 infection at 35 ± 2 °C (Table 4). Finally, the dichloromethane extracts led to a reduction from 6 Log10 PFU to 8 Log10 PFU (3.1 to 50 µg/mL) at 24 °C. The dichloromethane extract had the highest virucidal activity. Dichloromethane possesses an intermediate polar character between that of methanol and hexane: eluting forces of 0.26, 0.01, and 0.70, respectively.

### 3.5. PCA Analysis of the P. nurekis Extracts

The results of the multivariate analysis are presented in the Supplementary Materials Figure S5. There was a correlation between the carotenoid and polyphenol concentrations and the virucidal action of each extract, allowing differentiation between the extracts. The compounds with the greatest impact on antiviral activity were isobutyric acid and ethanol, followed by amino acids, such as L-aspartic acid, L-methionine, and glutamine. There was also a large impact of carotenoids, hypoxanthine, and organic acids, such as L-lactic, formic, pyruvic pyroglutamic, and succinic acid in the bioactivity of the samples against murine Coronavirus (Figure S5A,B). Amino acids other than those already mentioned, polyphenols, and alcohols other than ethanol had less of an impact on the antiviral activity against murine coronavirus.

The influence of carotenoids and phenols on the antiviral activity against herpesvirus (HSV-1) has already been reported for two green microalgae *Haematococcus pluvialis* and *Dunaliella salina*. The ethanol extract of *H. pluvialis*, a freshwater microalga, showed that such antiviral activity may be partially related to the presence of short-chain fatty acids. However, other compounds also contribute to this activity, such as those found in the ethanol extract of *D. salina*, a marine microalga. Further compounds may also be involved, such as β-ionone, neophytadiene, phytol, palmitic acid, and α-linolenic acid [67]. Ishikawa et al. [68] reported that β-carotene and astaxanthin, fucoxanthin, and the deacetylated metabolite fucoxanthinol had mild inhibitory effects on human T-cell leukaemia virus type 1-infected T-cell lines.

Our extracts could be differentiated by biomarkers, and it was possible to correlate the chemical composition with the antiviral activity. Previous studies have suggested that the antiviral activity against Coronavidae members can be attributed to metabolites such as phytol and polysaccharides [69], which we did not find in our study. Other researchers have reported a prominent role of organic acids in the viricidal effect, with one of the most prominent metabolites being formic acid, which can inhibit the replication of the porcine epidemic diarrhea virus (PEDV) [70]. According to the PCA, isobutyric acid was the metabolite with the highest impact on viricidal activity. It has already been reported that isobutyric acid and esters, such as 3-hydroxyisobutyrate, in combination with a low pH, generate an acidic environment that damages the structure of the virus and leads to viral inactivation. This mechanism has been observed for other compounds, such as acetic acid, but its effect is much weaker, which could explain why it showed a lower contribution to the antiviral activity against MHV-3 in the PCA. This mechanism is used by drugs, such as propylamylatin, against SARS-CoV-2, highlighting the potential application of *P. nurekis* extracts for the development of compounds against pathogens such as SARS-CoV-2 [71].

## 4. Conclusions

With the recent COVID-19 pandemic, research on coronavirus-inactivation mechanisms is urgently needed to find potential therapeutic agents against SARS-CoV-2. In this context, algae biotechnology has much to offer in the fight against coronaviruses. The use of algae is extremely promising due to its proven effectiveness against the virus when applied to animal cells and its low cost of production. Because it is a natural organism, it is not harmful to human beings or the environment. Here, we evaluated *P. nurekis* extracts obtained using three different solvents against MHV-3, a surrogate model for SARS-CoV-2. All the extracts efficiently inactivated MHV-3 (over 6 Log reductions), regardless of the solvent or concentration used. However, the dichloromethane extract showed the highest virucidal activity, demonstrating that this organic solvent of intermediate polarity is the most effective in extracting compounds with this bioactivity. As more metabolites become known, an increasingly detailed chemical profile will also become available, translating into a stronger correlation between the biological activity against SARS-CoV-2 and the compounds isolated from *P. nurekis* extracts. Based on multivariate analysis, polyphenols, carbohydrates, and isoprene derivatives, such as terpenic compounds and carotenoids, have a more significant impact on virucidal potential. More specific results are still needed to determine the precise correlation between the elucidated metabolites and the virucidal action of microalgae. Therefore, new tests on the L929 strain are recommended to evaluate the respiratory burst (NBT) and possible mechanisms of cellular damage, at the genetic level, as well as in vivo tests on MHV-3 in order to evaluate the activity of the extracts in the organism.

## Figures and Tables

**Table 1:** Concentration of the *Planktochlorella nurekis* extracts biomarkers and amino acids. Mean value and standard deviation (SD) (n = 3).

Biomarkers	Dichloromethane	Hexane	Methanol
Carotenoids (µg/g)	16.65 ± 3.04 ^a^	10.10 ± 0.91 ^b^	7.14 ± 0.49 ^c^
Polyphenols (mg/g)	40.96 ± 3.58 ^a^	29.17 ± 3.20 ^b^	84.91 ± 4.13 ^c^
Cysteine (mg/g)	15.94 ± 0.89 ^a^	16.21 ± 0.68 ^a^	15.67 ± 0.88 ^a^
Phenylalanine (mg/g)	30.06 ± 1.54 ^a^	30.52 ± 1.50 ^a^	29.59 ± 1.51 ^a^
Histidine (mg/g)	14.63 ± 1.23 ^a^	15.00 ± 0.94 ^a^	14.25 ± 1.21 ^a^
Isoleucine (mg/g)	164.54 ± 9.80 ^a^	167.48 ± 7.46 ^a^	161.58 ± 9.64 ^a^
Leucine (mg/g)	9.18 ± 2.05 ^a^	9.79 ± 1.56 ^a^	8.56 ± 2.02 ^a^
Proline (mg/g)	31.57 ± 3.08 ^a^	32.49 ± 2.34 ^a^	30.63 ± 3.03 ^a^
Serine (mg/g)	73.13 ± 6.15 ^a^	74.98 ± 6.15 ^a^	71.27 ± 6.05 ^a^
Threonine (mg/g)	99.13 ± 6.15 ^a^	100.98 ± 6.15 ^a^	97.27 ± 6.05 ^a^
Tryptophan (mg/g)	44.24 ± 2.05 ^a^	44.86 ± 1.56 ^a^	43.62 ± 2.02 ^a^
Valine (mg/g)	94.56 ± 6.94 ^a^	119.40 ± 5.29 ^b^	54.96 ± 6.83 ^c^

Values with similar letters do not present significant differences according to Tukey’s test (*p* < 0.05).

**Table 2:** Concentration of the *Planktochlorella nurekis* bioactives in NMR.

Compound Name	Concentration (µM)	
	Hexane Extract	Dichloromethane Extract
2-Hydroxybutyrate	0.3	11.1
3-Hydroxyisobutyrate	4.2	15.2
Acetic acid	0.2	0.6
Citric acid	0.8	10.2
Ethanol	0.7	0.7
Glycerol	0.1	N/A
Formic acid	0.9	11.4
L-Glutamic acid	1.0	1.1
Hypoxanthine	0.6	4.0
L-Tyrosine	0.1	1.1
L-Alanine	4.3	N/A
L-Threonine	1.6	12.1
L-Lactic acid	1.1	12.8
L-Aspartic acid	1.5	8.5
Pyruvic acid	N/A	1.2
Succinic acid	N/A	12.2
Pyroglutamic acid	N/A	6.7
3-Hydroxybutyric acid	0.6	2.0
Creatinine	3.7	N/A
L-Glutamine	N/A	13.3
L-Leucine	0.7	7.5
L-Methionine	N/A	1.9
L-Valine	9.6	14.3
Acetone	30.3	100.2
Isobutyric acid	19.0	17.8
1,5-Anhydrosorbitol	1.0	0.1
Dimethylsulfone	0.2	1.0
EDTA	0.1	N/A

**Table 3:** Cytotoxic evaluation of hexane, dichloromethane, and methanol extracts of *P. nurekis* using L929 cells. Mean value and standard deviation (SD).

Extracts	CC_50_^a^ µg/mL	CI_95_^b^ µg/mL
Hexane	330.4	270.1 to 404.3
Dichloromethane	53.19	30.94 to 91.45
Methanol	73.30	58.74 to 91.46

^a^ CC_50_—50% cytotoxic concentration; ^b^ CI_95_—95% confidence interval.

**Table 4:** Coronavirus—MHV-3—reduction (Log10) after treatment with hexane, dichloromethane, and methanol extracts of *P. nurekis*.

		Conc (µg/mL)				
Extract		3.12	6.25	12.5	25.00	50.00
Hexane	24 ± 2 °C	NR	NR	−7.0 ± 1.2	−7.0 ± 1.4	−7.0 ± 1.2
Dichloromethane		−6.0 ± 0.5	−6.0 ± 0.3	−6.0 ± 0.2	−8.0 ± 0.3	−8.0 ± 0.4
Methanol		−6.0 ± 1.5	−6.0 ± 0.5	−6.0 ± 0.8	−6.0 ± 0.3	−6.0 ± 0.4
Hexane	35 ± 2 °C	NR	NR	NR	NR	NR
Dichloromethane		NR	NR	−8.0 ± 0.3	−8.0 ± 0.3	−8.0 ± 0.4
Methanol		−6.0 ± 1.5	−6.0 ± 0.5	−6.0 ± 0.8	−6.0 ± 0.3	−6.0 ± 0.4

NR: no reduction.

## Supplementary Materials

The following supporting information can be downloaded at: https://www.mdpi.com/article/10.3390/ijerph192315823/s1, Supplementary Figure S1: Representative 1D 1H NMR spectra of the *Planktochlorella nurekis* extract in hexane (A), dichloromethane (B), and methanol (C).; Supplementary Figure S2: UPLC-ESIMS profile of the hexane extract; Supplementary Figure S3: UPLC-ESIMS profile of the DCM extract; Supplementary Figure S4: UPLC-ESIMS profile of the methanol extract; Supplementary Figure S5. PCA analysis of the *Planktochlorella nurekis* metabolites action against murine Coronavirus (a) Score plot *Planktochlorella nurekis* extracts (b). Loading plot of the and biomarkers and bioactivities. Supplementary Table S1: UPLC-ESIMS chemical composition of hexane, DCM, and methanol fractions of the algae.

## References

[1] Bárcena M., Oostergetel G.T., Bartelink W., Faas F.G.A., Verkleij A., Rottier P.J.M., Koster A.J., Bosch B.J. (2009). Cryo-electron tomography of mouse hepatitis virus: Insights into the structure of the coronavirion. Proc. Natl. Acad. Sci. USA.

[2] Peiris J.S.M. (2012). Coronaviruses. Medical Microbiology: Eighteenth Edition.

[3] Sharma H.B., Vanapalli K.R., Cheela V.S., Ranjan V.P., Jaglan A.K., Dubey B., Goel S., Bhattacharya J. (2020). Challenges, opportunities, and innovations for effective solid waste management during and post COVID-19 pandemic. Resour. Conserv. Recycl..

[4] Denis M., Vandeweerd V., Verbeke R., Version D.V. (2020). COVIPENDIUM: Information available to support the development of medical countermeasures and interventions against COVID-19 (Version 2020-05-19). Transdisciplinary In-Sights.

[5] Jiang F., Deng L., Zhang L., Cai Y., Cheung C.W., Xia Z. (2020). Review of the Clinical Characteristics of Coronavirus Disease 2019 (COVID-19). J. Gen. Intern. Med..

[6] Lee P.-I., Hsueh P.-R. (2020). Emerging threats from zoonotic coronaviruses-from SARS and MERS to 2019-nCoV. J. Microbiol. Immunol. Infect..

[7] Körner R.W., Majjouti M., Alcazar M.A.A., Mahabir E. (2020). Of Mice and Men: The Coronavirus MHV and Mouse Models as a Translational Approach to Understand SARS-CoV-2. Viruses.

[8] Züst R., Cervantes-Barragán L., Kuri T., Blakqori G., Weber F., Ludewig B., Thiel V. (2007). Coronavirus Non-Structural Protein 1 Is a Major Pathogenicity Factor: Implications for the Rational Design of Coronavirus Vaccines. PLoS Pathog..

[9] Gonzalez J.M., Gomez-Puertas P., Cavanagh D., Gorbalenya A., Enjuanes L. (2003). A comparative sequence analysis to revise the current taxonomy of the family Coronaviridae. Arch. Virol..

[10] Besednova N., Andryukov B., Zaporozhets T., Kryzhanovsky S., Fedyanina L., Kuznetsova T., Zvyagintseva T., Shchelkanov M. (2021). Antiviral Effects of Polyphenols from Marine Algae. Biomedicines.

[11] Abdo S.M., Hetta M.H., El-Senousy W.M., Salah El Din R.A., Ali G.H. (2012). Antiviral activity of freshwater algae. J. Appl. Pharm. Sci..

[12] Kim M., Yim J.H., Kim S.-Y., Kim H.S., Lee W.G., Kim S.J., Kang P.-S., Lee C.-K. (2012). In vitro inhibition of influenza A virus infection by marine microalga-derived sulfated polysaccharide p-KG03. Antivir. Res..

[13] Yim J.H., Kim S.J., Ahn S.H., Lee H.K. (2007). Characterization of a novel bioflocculant, p-KG03, from a marine dinoflagellate, Gyrodinium impudicum KG03. Bioresour. Technol..

[14] Shih S.-R., Tsai K.-N., Li Y.-S., Chueh C.-C., Chan E.-C. (2003). Inhibition of enterovirus 71-induced apoptosis by allophycocyanin isolated from a blue-green algaspirulina platensis. J. Med. Virol..

[15] O’Keefe B.R., Giomarelli B., Barnard D.L., Shenoy S.R., Chan P.K.S., McMahon J.B., Palmer K.E., Barnett B.W., Meyerholz D.K., Wohlford-Lenane C.L. (2010). Broad-Spectrum In Vitro Activity and In Vivo Efficacy of the Antiviral Protein Griffithsin against Emerging Viruses of the Family *Coronaviridae*. J. Virol..

[16] Rosales-Mendoza S., García-Silva I., González-Ortega O., Sandoval-Vargas J.M., Malla A., Vimolmangkang S. (2020). The Potential of Algal Biotechnology to Produce Antiviral Compounds and Biopharmaceuticals. Molecules.

[17] El-Baz F.K., El-Senousy W.M., El-Sayed A.B., Kamel M.M. (2013). In vitro antiviral and antimicrobial activities of Spirulina platensis extract. J. Appl. Pharm. Sci..

[18] Hasui M., Matsuda M., Okutani K., Shigeta S. (1995). In vitro antiviral activities of sulfated polysaccharides from a marine microalga (*Cochlodinium polykrikoides*) against human immunodeficiency virus and other enveloped viruses. Int. J. Biol.Macromol..

[19] Santoyo S., Plaza M., Jaime L., Ibañez E., Reglero G., Señorans F.J. (2010). Pressurized Liquid Extraction as an Alternative Process To Obtain Antiviral Agents from the Edible Microalga Chlorella vulgaris. J. Agric. Food Chem..

[20] Wang X., Zhang X. (2013). Separation, antitumor activities, and encapsulation of polypeptide from *Chlorella pyrenoidosa*. Biotechnol. Prog..

[21] Katharios P., Papadakis I.E., Prapas A., Dermon C.R., Ampatzis K., Divanach P. (2005). Mortality control of viral encephalopathy and retinopathy in 0+ grouper Epinephelus marginatus after prolonged bath in dense Chlorella minutissima culture. Bull. Eur. Assoc. Fish Pathol..

[22] Connan S., Stengel D.B. (2011). Impacts of ambient salinity and copper on brown algae: 2. Interactive effects on phenolic pool and assessment of metal binding capacity of phlorotannin. Aquat. Toxicol..

[23] Škaloud P., Němcová Y., Pytela J., Bogdanov N.I., Bock C., Pickinpaugh S.H. (2014). *Planktochlorella nurekis* gen. et sp. nov. (Trebouxiophyceae, Chlorophyta), a novel coccoid green alga carrying significant biotechnological potential. Fottea.

[24] Safafar H., Van Wagenen J.M., Møller P., Jacobsen C. (2015). Carotenoids, Phenolic Compounds and Tocopherols Contribute to the Antioxidative Properties of Some Microalgae Species Grown on Industrial Wastewater. Mar. Drugs.

[25] Aono Y., Asikin Y., Wang N., Tieman D., Klee H., Kusano M. (2021). High-Throughput Chlorophyll and Carotenoid Profiling Reveals Positive Associations with Sugar and Apocarotenoid Volatile Content in Fruits of Tomato Varieties in Modern and Wild Accessions. Metabolites.

[26] Rajeshwari K.R., Rajashekhar M. (2011). Biochemical composition of seven species of cyanobacteria isolated from different aquatic habitats of Western Ghats, Southern India. Braz. Arch. Biol. Technol..

[27] Vichai V., Kirtikara K. (2006). Sulforhodamine B colorimetric assay for cytotoxicity screening. Nat. Protoc..

[28] Pombal S., Hernández Y., Diez D., Mondolis E., Mero A., Morán-Pinzón J., Guerrero E.I., Rodilla J.M. (2017). Antioxidant activity of carvone and derivatives against superoxide ion. Nat. Prod. Commun..

[29] Tekiner M., Kurt M., Ak I., Kurt A. (2018). Determination of absorption coefficient of Chlorella vulgaris and Arthrospira maxima in water. AIP Conf. Proc..

[30] Lichtenthaler H.K., Buschmann C. (2001). Chlorophylls and Carotenoids: Measurement and Characterization by UV-VIS Spectroscopy. Curr. Protoc. Food Anal. Chem..

[31] Schmitz C., Pizzatto Dos Passos A., Bauer C.M., Cunha J., Mattioni B., Maraschin M. (2019). Comparison of five methods for lipid extraction from the Phaeodactylum tricornutum microalga and determination of fucoxanthin and fatty acids profiles. Adv. Biotechnol. Microbiol..

[32] Szpyrka E., Broda D., Oklejewicz B., Podbielska M., Slowik-Borowiec M., Jagusztyn B., Chrzanowski G., Kus-Liskiewicz M., Duda M., Zuczek J. (2020). A Non-Vector Approach to Increase Lipid Levels in the Microalga *Planktochlorella nurekis*. Molecules.

[33] Kumar G., Nguyen D.D., Sivagurunathan P., Kobayashi T., Xu K., Chang S.W. (2018). Cultivation of microalgal biomass using swine manure for biohydrogen production: Impact of dilution ratio and pretreatment. Bioresour. Technol..

[34] Hejazi M., Kleinegris D., Wijffels R. (2004). Mechanism of extraction of ?-carotene from microalgaDunaliellea salina in two-phase bioreactors. Biotechnol. Bioeng..

[35] Del Campo J.A., Rodríguez H., Moreno J., Vargas M.Á., Rivas J., Guerrero M.G. (2004). Accumulation of astaxanthin and lutein in *Chlorella zofingiensis* (Chlorophyta). Appl. Microbiol. Biotechnol..

[36] Craft N.E., Soares J.H. (1992). Relative solubility, stability, and absorptivity of lutein and.beta.-carotene in organic solvents. J. Agric. Food Chem..

[37] Barchan A., Bakkali M., Arakrak A., Pagán R., Laglaoui A. (2014). The effects of solvents polarity on the phenolic contents and antioxidant activity of three Mentha species extracts. Int. J. Curr. Microbiol. Appl. Sci..

[38] Boskou D., Tsimidou M., Blekas G. (2006). Polar Phenolic Compounds. Olive Oil: Chemistry and Technology: Second Edition.

[39] Pantami H., Bustamam M.A., Lee S., Ismail I., Faudzi S.M., Nakakuni M., Shaari K. (2020). Comprehensive GCMS and LC-MS/MS Metabolite Profiling of *Chlorella vulgaris*. Mar. Drugs.

[40] Nuzzo G., Gallo C., D’Ippolito G., Cutignano A., Sardo A., Fontana A. (2013). Composition and Quantitation of Microalgal Lipids by ERETIC 1H NMR Method. Mar. Drugs.

[41] Hussein H.A., Kassim M.N.I., Maulidiani M., Abas F., Abdullah M.A. (2022). Cytotoxicity and 1H NMR metabolomics analyses of microalgal extracts for synergistic application with Tamoxifen on breast cancer cells with reduced toxicity against Vero cells. Heliyon.

[42] Nzayisenga J., Sellstedt A. (2021). Metabolomic Study of Heterotrophically Grown *Chlorella* sp. Isolated from Wastewater in Northern Sweden. Molecules.

[43] Deyab M., Mofeed J., El-Bilawy E., Ward F. (2019). Antiviral activity of five filamentous cyanobacteria against coxsackievirus B3 and rotavirus. Arch. Microbiol..

[44] Marinho R.D.S.S., Ramos C.J.B., Leite J.P.G., Teixeira V.L., Paixão I.C.N.D.P., Belo C.A.D., Pereira A.B., Pinto A.M.V. (2016). Antiviral activity of 7-keto-stigmasterol obtained from green Antarctic algae Prasiola crispa against equine herpesvirus 1. J. Appl. Phycol..

[45] Zhang W., Tan N.G.J., Fu B., Li S.F.Y. (2015). Metallomics and NMR-based metabolomics of Chlorella sp. reveal the synergistic role of copper and cadmium in multi-metal toxicity and oxidative stress. Metallomics.

[46] Morales M., Collet P., Lardon L., Hélias A., Steyer J.-P., Bernard O. (2019). Life-cycle assessment of microalgal-based biofuel. Biofuels Algae.

[47] Chen H., Zheng Y., Zhan J., He C., Wang Q. (2017). Comparative metabolic profiling of the lipid-producing green microalga Chlorella reveals that nitrogen and carbon metabolic pathways contribute to lipid metabolism. Biotechnol. Biofuels.

[48] Carbone D., Pellone P., Lubritto C., Ciniglia C. (2021). Evaluation of Microalgae Antiviral Activity and Their Bioactive Compounds. Antibiotics.

[49] Romero-Martínez B.S., Montaño L.M., Solís-Chagoyán H., Sommer B., Ramírez-Salinas G.L., Pérez-Figueroa G.E., Flores-Soto E. (2021). Possible Beneficial Actions of Caffeine in SARS-CoV-2. Int. J. Mol. Sci..

[50] Yim S.-K., Kim I., Warren B., Kim J., Jung K., Ku B. (2021). Antiviral Activity of Two Marine Carotenoids against SARS-CoV-2 Virus Entry In Silico and In Vitro. Int. J. Mol. Sci..

[51] Ghasemnejad-Berenji M. (2021). Immunomodulatory and anti-inflammatory potential of crocin in COVID-19 treatment. J. Food Biochem..

[52] Al-Horani R.A., Kar S. (2020). Potential Anti-SARS-CoV-2 Therapeutics That Target the Post-Entry Stages of the Viral Life Cycle: A Comprehensive Review. Viruses.

[53] Afreen R., Tyagi S., Singh G.P., Singh M. (2021). Challenges and Perspectives of Polyhydroxyalkanoate Production From Microalgae/Cyanobacteria and Bacteria as Microbial Factories: An Assessment of Hybrid Biological System. Front. Bioeng. Biotechnol..

[54] Azizan A., Bustamam M.S.A., Maulidiani M., Shaari K., Ismail I.S., Nagao N., Abas F. (2018). Metabolite Profiling of the Microalgal Diatom Chaetoceros Calcitrans and Correlation with Antioxidant and Nitric Oxide Inhibitory Activities via 1H NMR-Based Metabolomics. Mar. Drugs.

[55] Sarpal A.S., Teixeira C.M.L.L., Silva P.R.M., Monteiro T.V.D.C., da Silva J.I., da Cunha V.S., Daroda R.J. (2015). NMR techniques for determination of lipid content in microalgal biomass and their use in monitoring the cultivation with biodiesel potential. Appl. Microbiol. Biotechnol..

[56] Davey P.T., Hiscox W.C., Lucker B.F., O’Fallon J.V., Chen S., Helms G.L. (2012). Rapid triacylglyceride detection and quantification in live micro-algal cultures via liquid state 1H NMR. Algal Res..

[57] Guella G., Frassanito R., Mancini I. (2003). A new solution for an old problem: The regiochemical distribution of the acyl chains in galactolipids can be established by electrospray ionization tandem mass spectrometry. Rapid Commun. Mass Spectrom..

[58] Barbosa M., Valentão P., Andrade P.B. (2016). Biologically Active Oxylipins from Enzymatic and Nonenzymatic Routes in Macroalgae. Mar. Drugs.

[59] Maltsev Y., Maltseva K. (2021). Fatty acids of microalgae: Diversity and applications. Rev. Environ. Sci. Bio/Technology.

[60] Kristjánsson J.M., Rolfsson Ó. (2021). Virucidal activity of a proprietary blend of plant-based oils (Viruxal) against SARS-CoV-2 and influenza viruses—An in vitro study. bioRxiv.

[61] Mofeed J., Deyab M., Mohamed A., Moustafa M., Negm S., El-Bilawy E. (2022). Antimicrobial activities of three seaweeds extract against some human viral and bacterial pathogens. BIOCELL.

[62] Ferdous U.T., Yusof Z.N.B. (2021). Insight into Potential Anticancer Activity of Algal Flavonoids: Current Status and Challenges. Molecules.

[63] Ferreira G.F., Pessoa J.G.B., Pinto L.F.R., Filho R.M., Fregolente L.V. (2021). Mono- and diglyceride production from microalgae: Challenges and prospects of high-value emulsifiers. Trends Food Sci. Technol..

[64] Nascimento T.C.D., Pinheiro P.N., Fernandes A.S., Murador D.C., Neves B.V., de Menezes C.R., de Rosso V.V., Jacob-Lopes E., Zepka L.Q. (2020). Bioaccessibility and intestinal uptake of carotenoids from microalgae Scenedesmus obliquus. LWT.

[65] Banskota A.H., Stefanova R., Gallant P., Osborne J.A., Melanson R., O’Leary S.J. (2013). Nitric oxide inhibitory activity of monogalactosylmonoacylglycerols from a freshwater microalgae *Chlorella sorokiniana*. Nat. Prod. Res..

[66] Solinski A.E., Koval A.B., Brzozowski R.S., Morrison K.R., Fraboni A.J., Carson C.E., Eshraghi A.R., Zhou G., Quivey R.G., Voelz V.A. (2017). Diverted Total Synthesis of Carolacton-Inspired Analogs Yields Three Distinct Phenotypes in Streptococcus mutans Biofilms. J. Am. Chem. Soc..

[67] Santoyo S., Jaime L., Plaza M., Herrero M., Rodriguez-Meizoso I., Ibañez E., Reglero G. (2012). Antiviral compounds obtained from microalgae commonly used as carotenoid sources. J. Appl. Phycol..

[68] Ishikawa C., Tafuku S., Kadekaru T., Sawada S., Tomita M., Okudaira T., Nakazato T., Toda T., Uchihara J.-N., Taira N. (2008). Antiadult T-cell leukemia effects of brown algae fucoxanthin and its deacetylated product, fucoxanthinol. Int. J. Cancer.

[69] Reynolds D., Huesemann M., Edmundson S., Sims A., Hurst B., Cady S., Beirne N., Freeman J., Berger A., Gao S. (2021). Viral inhibitors derived from macroalgae, microalgae, and cyanobacteria: A review of antiviral potential throughout pathogenesis. Algal Res..

[70] Gómez-García M., Puente H., Argüello H., Mencía-Ares Ó., Rubio P., Carvajal A. (2021). In vitro Assessment of Antiviral Effect of Natural Compounds on Porcine Epidemic Diarrhea Coronavirus. Front. Veter Sci..

[71] Brown A., Strobel G., Hanrahan K., Sears J. (2021). Antiviral Activity of the Propylamylatin^TM^ Formula against the Novel Coronavirus SARS-CoV-2 In Vitro using direct injection and gas assays in Virus suspensions. Viruses.

