# Supplementary material for: Characterization of Planktochlorella nurekis Extracts and Virucidal Activity against a Coronavirus Model, the Murine Coronavirus 3
Source: Int J Environ Res Public Health. 2022 Nov 28;19(23):15823. doi: 10.3390/ijerph192315823 (PMC9735810; doi:10.3390/ijerph192315823)
Supplement: Supplementary file 1 [file ijerph-19-15823-s001.zip › ijerph-2004911-supplementary.pdf]

# Supplementary Materials

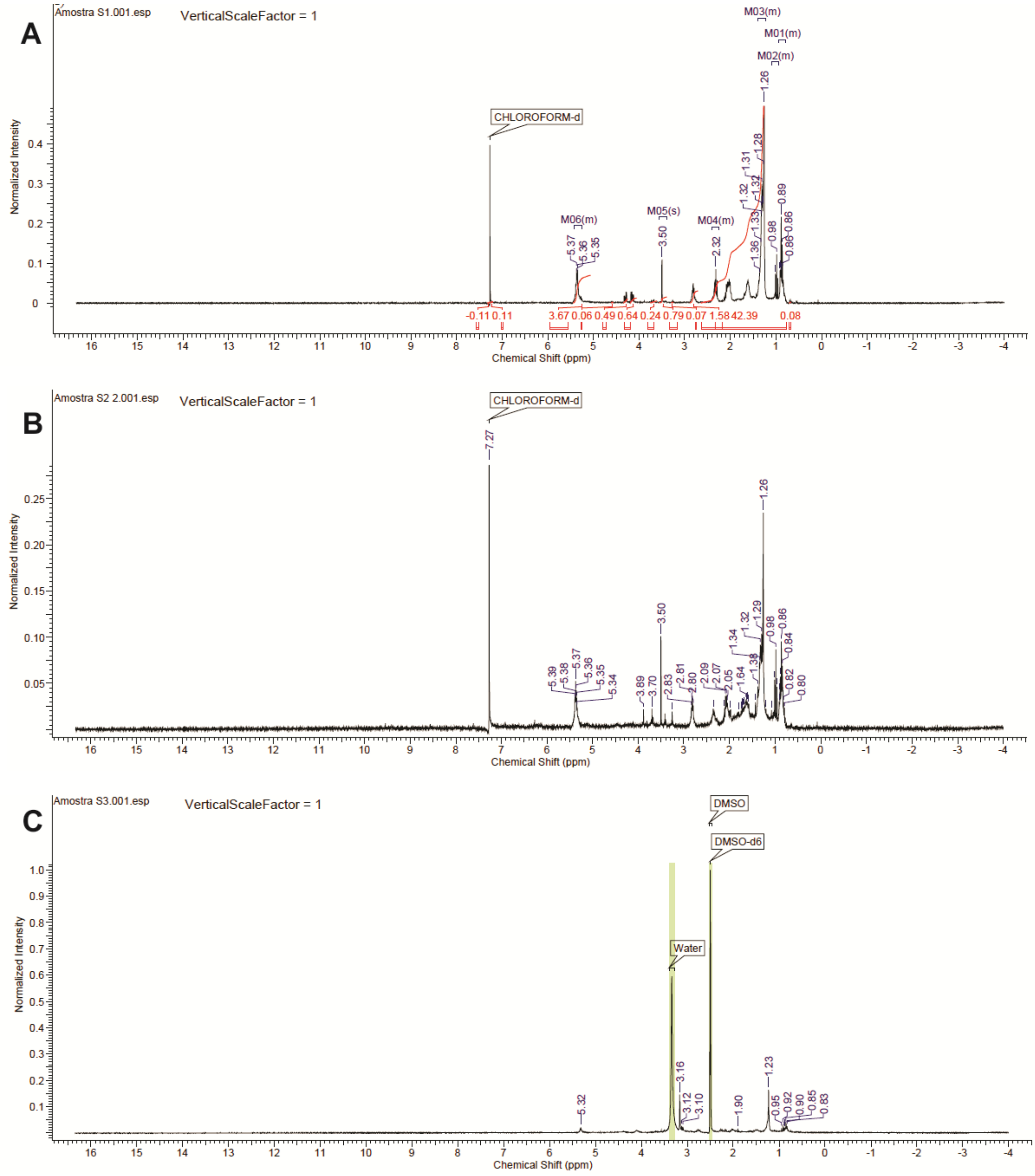


**Figure S1**. Representative 1D $^{1}$H NMR spectra of the *Planktochlorella nurekis* extract in hexane (A), dichlorometane (B), and methanol (C).

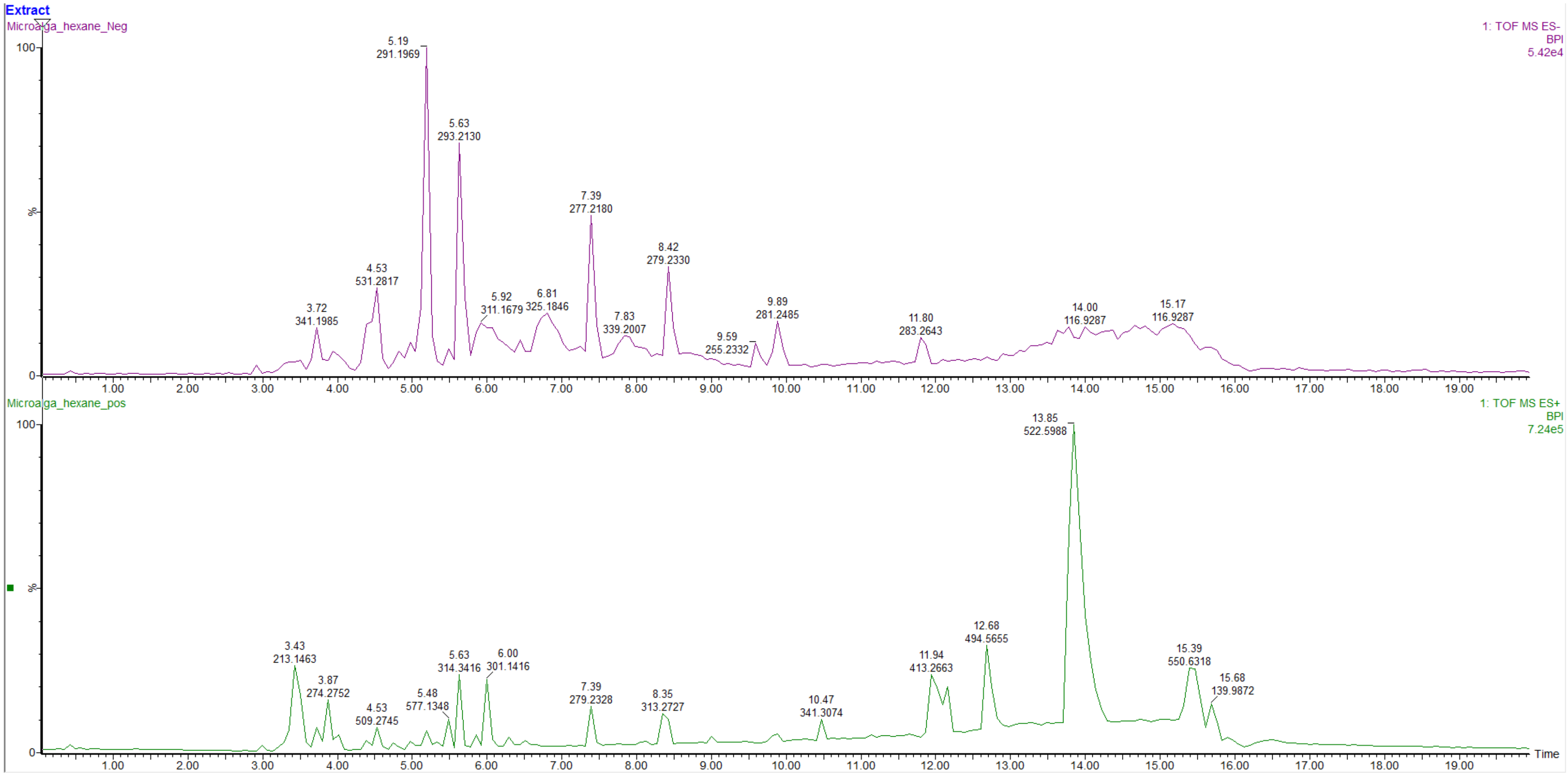


**Figure S2**. UPLC-ESIMS profile of the hexane extract.

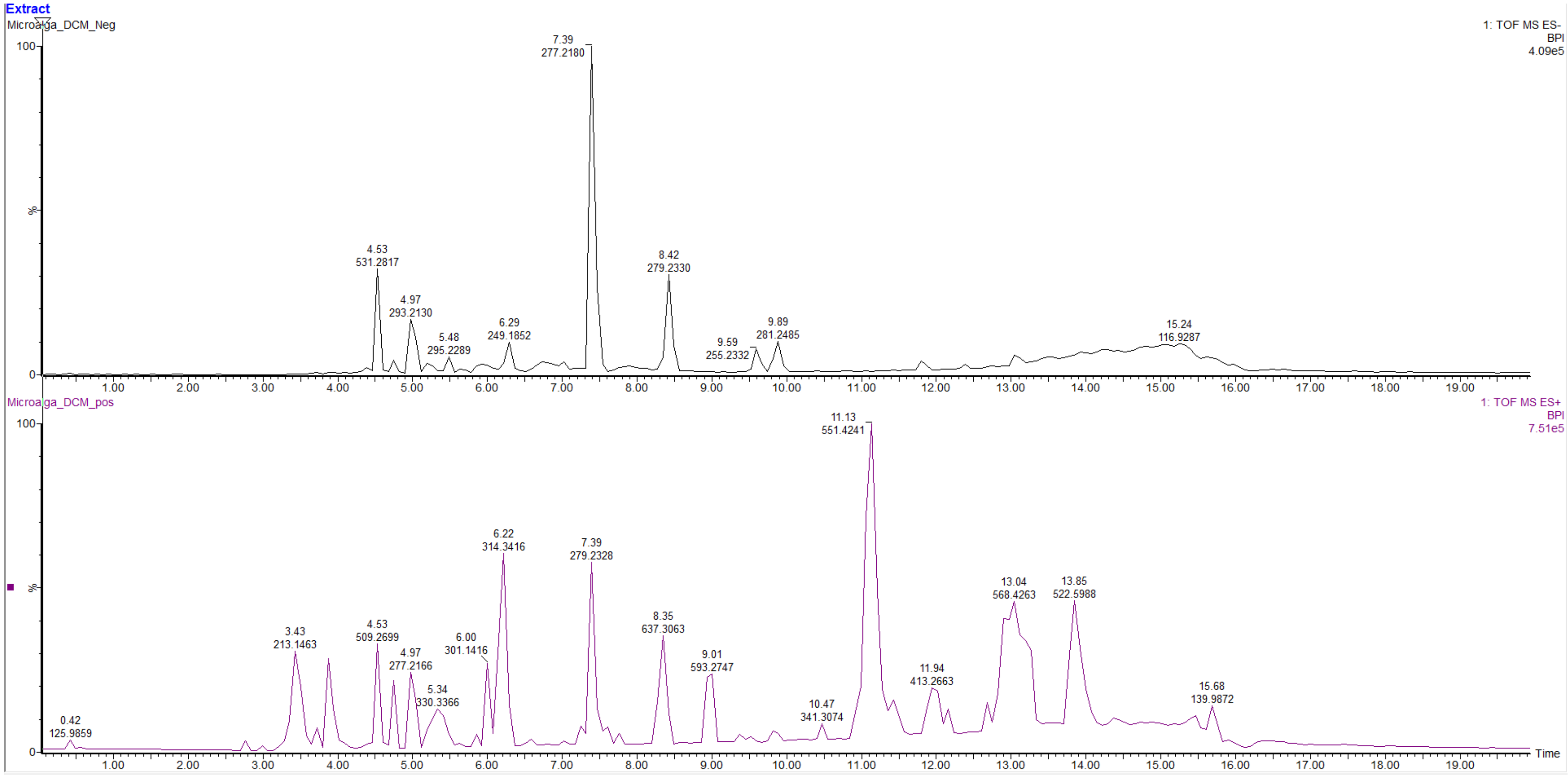


**Figure S3**. UPLC-ESIMS profile of the DCM extract.

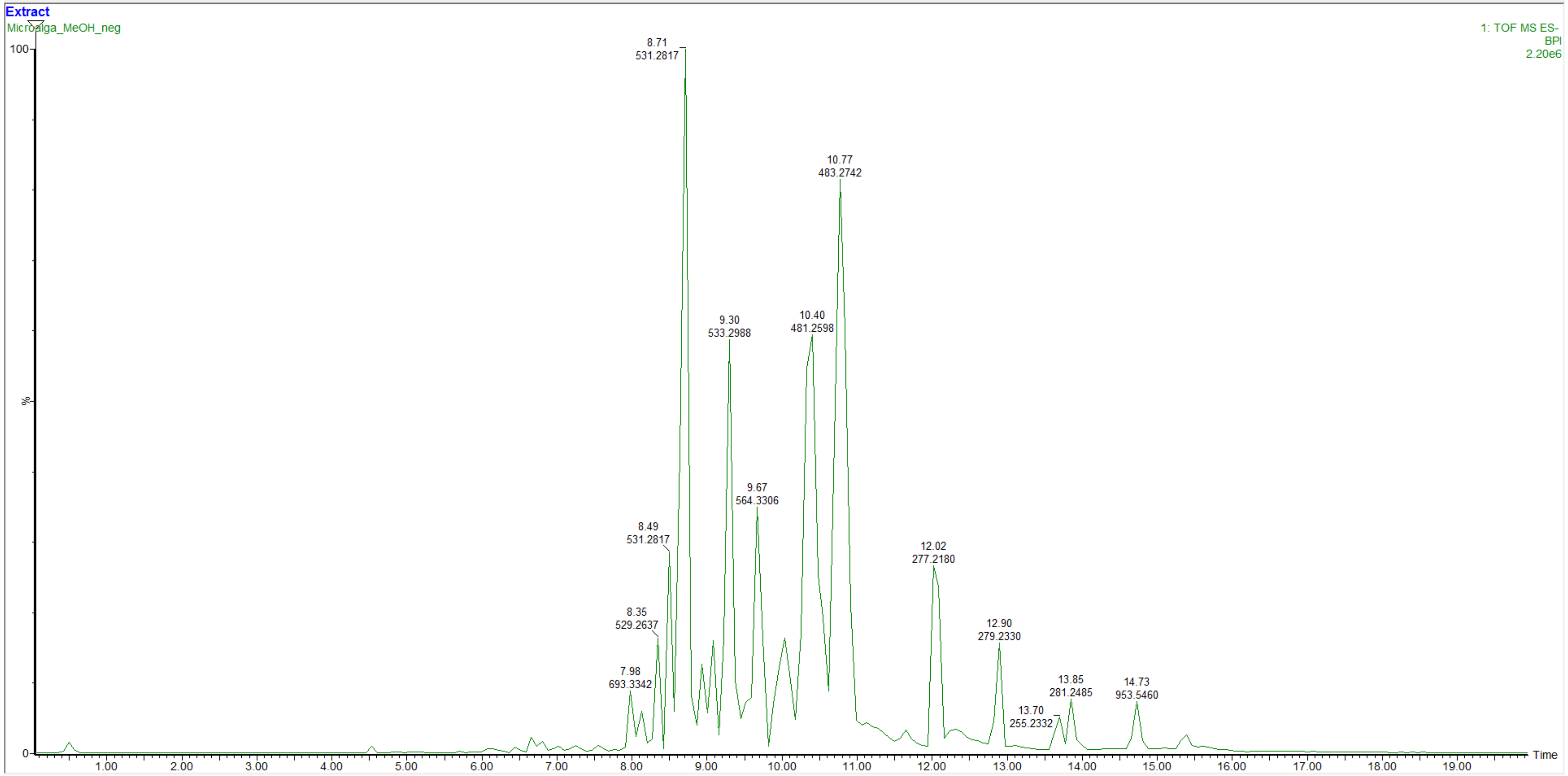


**Figure S4**. UPLC-ESIMS profile of the methanol extract.

**Table S1**. UPLC-ESIMS chemical composition of hexane, DCM, and methanol fractions of the algae.

| | tR | Peak (m/z) and MF | Error | Fragment ions (m/z) | Proposed structures | Hexane | DCM |
|---|---|---|---|---|---|---|---|
| ESI-MS-(-) | | | | | | Extract | |
| 1 | 4.53 | 531.2817 [$C_{25}H_{42}O_9$+$HCO_2$]$^-$ 485.2748 [$C_{25}H_{42}O_9$-H]$^-$ | 2.19 -0.53 | - | (7Z,10Z,13Z)-2-hydroxy-3-(β-D-galactopyranosyl)oxy)propyl hexadeca-7,10,13-trienoate | X | X |
| 2 | 4.97 | 293.2130 [$C_{18}H_{30}O_3$-H]$^-$ | 4.54 | 275.2035 | 13-hydroxyoctadecatrienoic acid | X | X |
| 3 | 5.48 | 295.2289 [$C_{18}H_{32}O_3$-H]$^-$ | 5.34 | - | Vernolic acid | - | X |
| 4 | 6.29 | 249.1852 [$C_{16}H_{26}O_2$-H]$^-$ | -1.02 | - | Hexadecatrienoic acid | - | X |
| 5 | 7.39 | 277.2180 [$C_{18}H_{30}O_2$-H]$^-$ | 4.49 | - | Linolenic acid | X | X |
| 6 | 8.42 | 279.2330 [$C_{18}H_{32}O_2$-H]$^-$ | 2.13 | - | Linoleic acid | X | X |
| 7 | 9.59 | 255.2332 [$C_{16}H_{32}O_2$-H]$^-$ | 3.11 | - | Palmitic acid | - | X |
| 8 | 9.89 | 281.2485 [$C_{18}H_{34}O_2$-H]$^-$ | 1.58 | | Oleic acid | X | X |
| ESI-MS-(+) | | | | | | | |
| 9 | 3.72 | 181.1233 [$C_{11}H_{16}O_2$+H]$^+$ | 2.46 | 163.1130 | 4,4,7a-trimethyl-5,6,7,7a-tetrahydrobenzofuran-2(4H)-one | - | X |
| 1 | 4.53 | 509.2745 [$C_{25}H_{42}O_9$+Na]$^+$ | 3.63 | 469.2790, 325.2374, 233.1918 | (7Z,10Z,13Z)-2-hydroxy-3-(β-D-galactopyranosyl)oxy)propyl hexadeca-7,10,13-trienoate | X | X |
| 10 | 4.97 | 277.2166 [$C_{18}H_{28}O_2$+H]$^+$ | -0,56 | | Stearidonic acid | - | X |
| 11 | 5.48 | 577.1348 [$C_{30}H_{24}O_{12}$+H]$^+$ | 0.34 | 385.0938 | Related to proanthocyanidin A1 | X | - |
| 12 | 6.00 | 301.1416 [$C_{16}H_{22}O_{4+}$Na]$^+$ | 0.07 | 205.0866, 149.0244 | Dibutyl phthalate | X | X |
| 5 | 7.39 | 279.2328 [$C_{18}H_{30}O_2$+H]$^+$ | 1.41 | - | Linolenic acid | X | X |

| | | | | | | | |
|---|---|---|---|---|---|---|---|
| 13 | 8.35 | 637.3063 $[C_{29}H_{48}O_{15}+H]^+$ | -1.33 | 581.2450, 525.1830, 495.26262, 469.1125 | Derivative of O-tetrapropanoyloctanoate of sucrose | - | X |
| 14 | 8.35 | 331.2852 $[C_{19}H_{38}O_4+H]^+$ | 1.10 | 313.2727, 239.2368 | monopalmitin | X | - |
| 15 | 9.01 | 593.2747 $[C_{34}H_{40}O_9+H]^+$ | -0.6 | 533.2573 | unidentified | - | X |
| 16 | 10.47 | 359.3179 $[C_{21}H_{42}O_4+H]^+$ | 4.91 | 341.3074, 267.2713 | monostearin | X | X |
| 17 | 11.13 | 551.4241 $[C_{40}H_{54}O+H]^+$ | -2.16 | - | Echinenone | - | X |
| 18 | 11.43 | 607.2911 $[C_{33}H_{44}O_9+Na]^+$ | 4.61 | 547.2725 | unidentified | - | X |
| 19 | 11.94 | 413.2663 $[C_{24}H_{38}O_4+Na]^+$ | -1.16 | 301.1410, 149.0244 | Dioctyl phthalate | X | X |
| Methanol Extract - ESI-MS-(-) | | | | | | | |
| 20 | 7.98 | 693.3342 $[C_{31}H_{52}O_{14}+HCO_2]^-$ | 1.21 | 647.3315$[M-H]^-$, 249.1852 | 3-hydroxy-2-[[(7Z,10Z,13Z)-1-oxo-7,10,13-hexadecatrienyl]oxy]propyl 6-O-β-D-galactopyranosyl-β-D-Galactopyranoside | | |
| 21 | 8.35 | 529.2637 $[C_{25}H_{40}O_9+HCO_2]^-$ | -2.24 | 483.2607 $[M-H]^-$, 247.1671 | 3-hydroxy-2-[[-1-oxo-4,7,10,13-hexadecatetraenyl]oxy]propyl β-D-Galactopyranoside | | |
| 22 | 8.49 | 531.2817 $[C_{25}H_{42}O_9+HCO_2]^-$ | 2.19 | 485.2748, 249.1852 | 3-hydroxy-2-[[-1-oxo-7,10,13-hexadecatrienyl]oxy]propyl β-D-Galactopyranoside | | |
| 23 | 8.71 | 531.2817 $[C_{25}H_{42}O_9+HCO_2]^-$ | 2.19 | 485.2748, 249.1852 | Isomer of compound 22 | | |
| 24 | 8.93 | 721.3671 $[C_{33}H_{56}O_{14}+HCO_2]^-$ | 3.38 | 675.3586, 277.2180 | 2-hydroxy-3-[[(9Z,12Z,15Z)-1-oxo-9,12,15-octadecatrien-1-yl]oxy]propyl 6-O-β-D-galactopyranosyl-β-D-Galactopyranoside, | | |

| | | | | | |
|---|---|---|---|---|---|
| 25 | 9.30 | 533.2988 [$C_{25}H_{44}O_9$+$HCO_2$]$^-$ | 4.90 | 487.2931, 251.2029 | (2S)-2-hydroxy-3-[[(7Z,10Z)-1-oxo-7,10-hexadecadien-1-yl]oxy]propyl β-D-Galactopyranoside |
| 26 | 10.03 | 699.3804 [$C_{31}H_{58}O_{14}$+$HCO_2$]$^-$ | 0.13 | 255.2332 | 1−O−palmitoyl−3−O−[α−D−galactopyranosyl (1→6)−β−D−galactopyranosyl]−sn−glycerol |
| 27 | 10.40 | 481.2598 [$C_{29}H_{38}O_6$-H]$^-$ | 1.63 | 281.2485, 255.2332 | Steroid |
| 28 | 10.77 | 483.2742 [$C_{29}H_{40}O_6$-H]$^-$ | -0.96 | 255.2332 | Steroid |
| 29 | 12.02 | 277.2180 [$C_{18}H_{30}O_2$-H]$^-$ | 4.49 | - | Linolenic acid derivative |
| 30 | 12.90 | 279.2330 [$C_{18}H_{32}O_2$-H]$^-$ | 2.13 | - | Linoleic acid derivative |
| 31 | 13.70 | 255.2332 [$C_{16}H_{32}O_2$-H]$^-$ | 3.11 | - | Palmitic acid derivative |
| 32 | 13.85 | 281.2485 [$C_{18}H_{34}O_2$-H]$^-$ | 1.58 | | Oleic acid derivative |
| 33 | 14.73 | 953.5460 [$C_{49}H_{80}O_{15}$+$HCO_2$]$^-$ | -1.44 | 907.5478 [M-H]$^-$, 249.1852, 277.2180 | [[-1-oxo-9-hexadecatrien-1-yl]oxy]-3-[[-1-oxo-octadecatrien-1-yl]oxy]propyl 6-O-α-D-galactopyranosyl-β-D-Galactopyranoside |

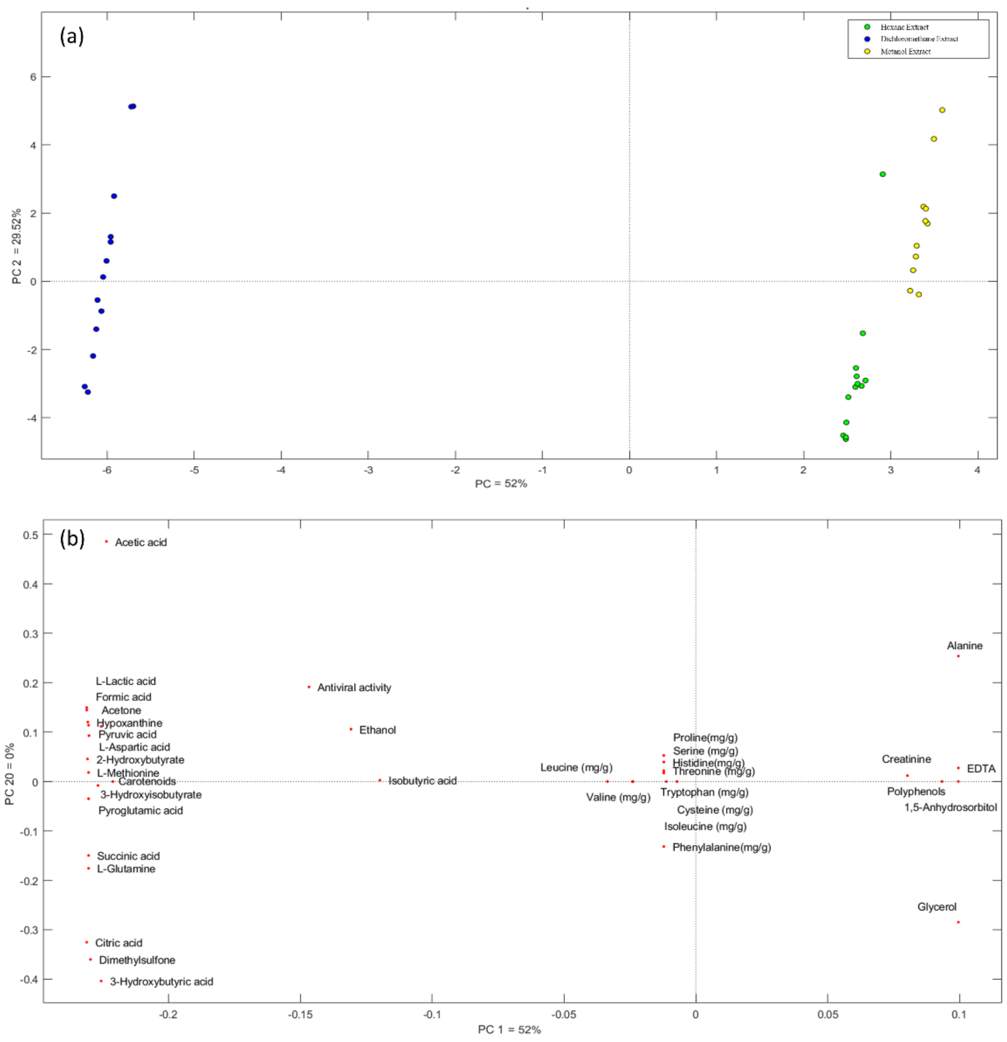


**Figure S5**. PCA analysis of the *Planktochlorella nurekis* metabolites action against murine Coronavirus (a) Score plot *Planktochlorella nurekis* extracts (b). Loading plot of the and biomarkers and bioactivities.
